# Dissimilar Reactions and Enzymes for Psilocybin Biosynthesis in *Inocybe* and *Psilocybe* Mushrooms

**DOI:** 10.1002/anie.202512017

**Published:** 2025-09-21

**Authors:** Tim Schäfer, Fabian Haun, Bernhard Rupp, Dirk Hoffmeister

**Affiliations:** ^1^ Pharmaceutical Microbiology Friedrich Schiller University Winzerlaer Str. 2 07745 Jena Germany; ^2^ Pharmaceutical Microbiology Leibniz Institute for Natural Product Research and Infection Biology – Hans‐Knöll‐Institute Beutenbergstrasse 11a 07745 Jena Germany; ^3^ Department of General, Inorganic and Theoretical Chemistry University of Innsbruck Innrain 82 6020 Innsbruck Austria; ^4^ k.k. Hofkristallamt San Diego California USA; ^5^ Cluster of Excellence Balance of the Microverse Friedrich Schiller University Neugasse 23 07743 Jena Germany

**Keywords:** Decarboxylase, *Inocybe*, Kinase, Methyltransferase, Psilocybin

## Abstract

Psilocybin (4‐phosphoryloxy‐*N*,*N*‐dimethyltryptamine, **1**) is the main indolethyl‐amine natural product of psychotropic (so‐called “magic”) mushrooms. The majority of **1**‐producing species belongs to the eponymous genus *Psilocybe*, for which the biosynthetic events, beginning from l‐tryptophan (**2**), and the involved enzymes have thoroughly been characterized. Some *Inocybe* (fiber cap) species, among them *Inocybe corydalina*, produce **1** as well. In product formation assays, we characterized four recombinantly produced biosynthesis enzymes of this species in vitro: IpsD, a pyridoxal‐5′‐phosphate‐dependent l‐tryptophan decarboxylase, the kinase IpsK, and two near‐identical methyltransferases, IpsM1 and IpsM2. The fifth enzyme, the insoluble monooxygenase IpsH, was analyzed in silico. Surprisingly, none of the reactions intrinsic to the **1** pathway in *Psilocybe* species takes place in *I. corydalina*. Contrasting the situation in *Psilocybe*, the *Inocybe* pathway is branched and leads to baeocystin (4‐phosphoryloxy‐*N*‐methyltryptamine, **3**) as a second end product. Our results demonstrate that mushrooms recruited distantly or entirely unrelated enzymes to evolve the metabolic capacity for **1** biosynthesis twice independently.

## Graphical abstract

Mushrooms have learned twice independently how to make the iconic magic mushroom natural product psilocybin. This article introduces the enzymes of the second pathway, found in a fiber cap mushroom. Curiously, the two pathways do not share any reaction, nor do the enzymes show a close relationship, but both pathways proceed via 4‐hydroxytryptamine as a common intermediate.

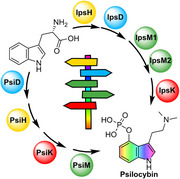

## Introduction

**Scheme 1:**
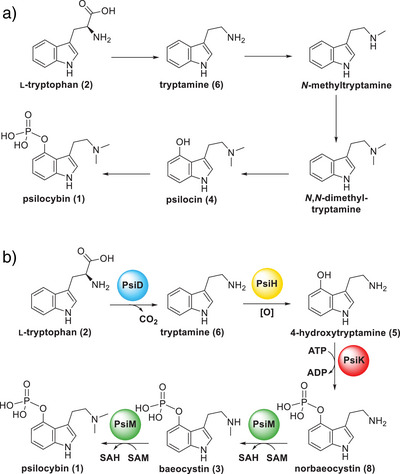
Biosynthesis of **1** in *Psilocybe cubensis*. Compound **2** as origin was proposed by Hofmann et al.^[^ ^5^ ^]^ a) Sequence proposed in 1968 by Agurell and Nilsson based on ^14^C and ^3^H radiotracer incorporation.^[^ ^6^ ^]^ b) Sequence proposed in 2017 based on characterized biosynthesis enzymes.^[^ ^7^ ^]^

The principal natural product of “magic” mushrooms is psilocybin (**1**, Scheme 1),^[^ ^1^, ^2^ ^]^ a 4‐*O*‐phosphorylated indolethyl‐amine and chemically stable precursor of its dephosphorylated analog psilocin (**4**, Scheme 1). The latter—chemically unstable—compound represents the actual psychotropic compound interfering with serotonergic neurotransmission by binding to 5‐hydroxytryptamine (5‐HT) receptors, mainly the 5‐HT_2A_ receptor, with high affinity.^[^ ^3^ ^]^ The pharmaceutical value of **1** roots in its status as a candidate drug against severe and therapy‐refractory depression, with promising outcomes in advanced clinical trials.^[^ ^4^ ^]^

**Figure 1:**
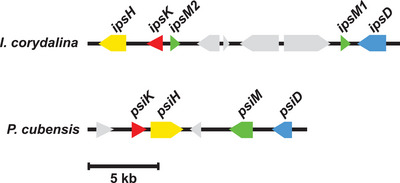
Clusters of genes encoding **1** biosynthetic enzyme in *Inocybe corydalina* (*ips* genes, upper map) and *Psilocybe cubensis* (*psi* genes, lower map). The clusters each code for a kinase (*ipsK* and *psiK*, red), for a P450 monooxygenase (*ipsH* and *psiH*, yellow), for two methyltransferases (*ipsM1* and *ipsM2*, green) in *I. corydalina* and one in *P. cubensis* (*psiM*), as well as for a tryptophan decarboxylase (*ipsD* and *psiD*, blue). Hypothetical genes are shown in gray. For clarity, introns are not shown.

In pioneering work by pharmaceutical chemist Hofmann and his coworkers, **1**, and in lower amounts **4**, were isolated from fruiting bodies of *Psilocybe* (*P*.) *mexicana*, and the biosynthetic origin from l‐tryptophan (**2**) was shown.^[^ ^1^, ^2^, ^5^ ^]^ Furthermore, the regioselective 4‐hydroxylation of the indole nucleus was postulated as the initial biosynthetic event.^[^ ^5^ ^]^ Based on ^14^C and ^3^H radiotracer studies in *P*. *cubensis*, Agurell and Nilsson subsequently proposed a cascade from **2** to **4** before a phosphate ester formation completes **1** biosynthesis (Scheme 1a).^[^ ^6^ ^]^ The discovery of the *psi* genes in various *Psilocybe* and *Panaeolus* species (Figure 1),^[^ ^7^, ^8^, ^9^ ^]^ allowed for in‐depth characterization of the biosynthetic enzymes^[^ ^10^, ^11^, ^12^, ^13^ ^]^ and culminated in a biochemically proven sequence of how this iconic natural product is assembled by *P. cubensis* (Scheme 1b).^[^ ^7^ ^]^

Remarkably, this sequence excludes both dimethyltryptamine (which could be hydroxylated to **4** by PsiH) and **4** itself as intermediates. Furthermore, PsiK phosphorylates **4** to **1** with a higher catalytic efficiency than its actual substrate, 4‐hydroxytryptamine (**5**).^[^ ^11^ ^]^ Taken together, these findings suggest a biosynthetic strategy that keeps the producing cells clear of **4**, and experimental evidence exists that **4** is an artifact due to the work‐up of biomass rather than a *Psilocybe* natural product.^[^ ^14^ ^]^ This pathway—established experimentally in vitro—has been validated multiple times by heterologous reconstitution in vivo, first in an *Aspergillus* mold,^[^ ^15^, ^16^ ^]^ followed by yeast^[^ ^17^ ^]^ and *Escherichia coli*.^[^ ^18^, ^19^, ^20^ ^]^ While **1** has traditionally been most closely associated with the eponymous mushroom genus *Psilocybe*, the compound and the genes were detected in species of other genera as well, among them *Gymnopilus*, *Panaeolus*, and *Pluteus*.^[^ ^8^, ^21^, ^22^, ^23^ ^]^ The identification of the *psi* genes was therefore key to tracing the evolution of this pathway and its distribution across genera by various horizontal gene transfers.^[^ ^8^, ^21^, ^22^ ^]^

The mushroom genus *Inocybe* (the fiber caps in the traditional circumscription of the genus) is best known for its fatal l‐(+)‐muscarine‐producing species. Yet, **1** was predicted to occur in *Inocybe* (*I*.) *aeruginascens* as early as 1983,^[^ ^24^ ^]^ and was subsequently detected in this and other *Inocybe* species, among them *I. corydalina*.^[^ ^25^, ^26^, ^27^ ^]^ Furthermore, phylogenetic analyses on the family Inocybaceae demonstrated that l‐(+)‐muscarine and **1** occur mutually exclusively.^[^ ^28^ ^]^ Intriguingly, Awan et al. reported that *I. corydalina* genomic DNA does not encode *psi* genes. Rather, a cluster of genes unrelated to the *psi* gene cluster, yet putatively encoding enzymes that carry out the same catalytic functions, was hypothesized to confer the capacity to produce **1**.^[^ ^23^ ^]^

We followed up on this hypothesis and report the in vitro biochemical characterization and in silico modeling of heterologously produced *I. corydalina* **1** biosynthesis enzymes. Our results prove a deviating biosynthetic sequence, compared to *Psilocybe* species, in which none of the reactions in one pathway occur in the other. We provide biochemical evidence that **1** biosynthesis enzyme in *Inocybe* is dissimilar to those familiar from *Psilocybe*, even though both genera belong to the same phylogenetic order of mushrooms, the Agaricales. Furthermore, our results expand the repertoire of catalysts suitable to produce **1** biotechnologically in vivo^[^ ^15^, ^16^, ^17^, ^18^, ^19^, ^20^ ^]^ or in vitro^[^ ^29^ ^]^ as a future drug.

## Results and Discussion

In 2018, Awan et al. presented the genomic DNA of *I. corydalina* and five co‐localized genes that putatively encode enzymes that confer all activities required to catalyze **1** formation from **2**.^[^ ^23^ ^]^ These genes, hereafter referred to as *ips* genes, were predicted to encode an aromatic amino acid decarboxylase IpsD, a monooxygenase IpsH, a kinase IpsK, and two near‐identical *S*‐adenosyl‐l‐methionine (SAM)‐dependent methyltransferases, IpsM1 and IpsM2 (Figure 1). Collectively, these activities catalyze **1** biosynthesis in numerous *Psilocybe* species. However, following the phylogenetic characterization by Awan et al., the *I. corydalina* enzymes do not share a close common ancestry with the confirmed Psi enzymes of *Psilocybe* species.

### Activity and Structural Model of the Decarboxylase IpsD

**Figure 2:**
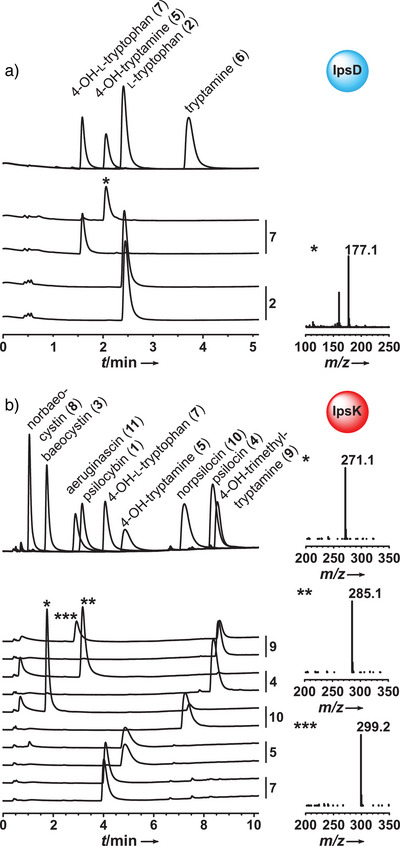
Chromatographic analysis with UV detection (λ = 280 nm) of in vitro product formation assays. Authentic standards are shown as overlaid individual chromatograms. a) Decarboxylation assays with IpsD, pairs of chromatograms are designated with the respective substrate (**7** or **2**). b) Phosphotransfer assays with IpsK. As above, pairs of chromatograms are designated with the substrate (**9**, **4**, **10**, **5**, and **7**). Reactions are shown in Scheme 2. The bottom traces of each pair of chromatograms represent negative controls with heat‐treated enzymes. Insets marked with asterisks depict mass spectra of the respective peak. Mass spectra were recorded in positive mode.

**Scheme 2:**
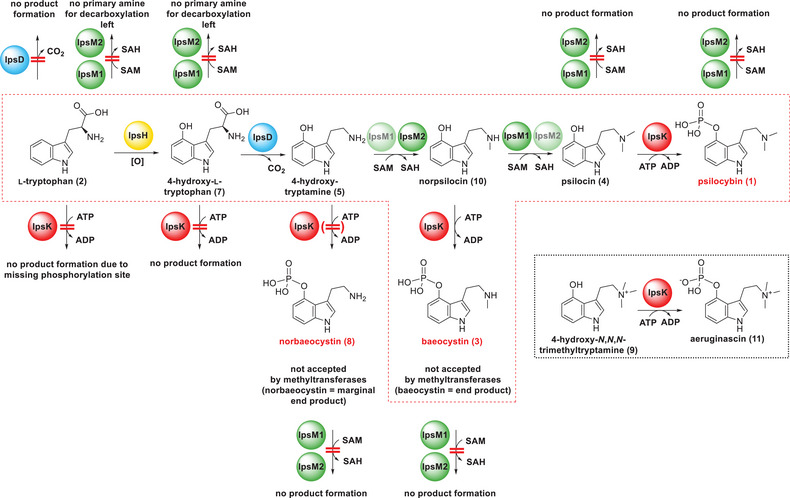
Biosynthetic sequence to **1** in *I. corydalina*. The experimentally proven pathway to **1** and its branch to baeocystin (**3**) as a major product (dashed red frame) and to norbaeocystin (**8**) as a trace product are shown. The reaction catalyzed by IpsH is deduced from the substrate specificities of the other enzymes. Also shown are routes that are unfeasible out of theoretical considerations or due to the enzymes’ substrate specificities.

BlastP analysis of the IpsD amino acid sequence (Table S1) shows a standard binding pocket for the prosthetic group pyridoxal‐5′‐phosphate (PLP). Native IpsD shares 63% identical amino acids (aa) with the aromatic amino acid decarboxylase CsTDC of the mushroom *Gelatiporia subvermispora*.^[^ ^30^ ^]^ In contrast, PsiD, the gateway enzyme of **1** formation in *Psilocybe* species, is phylogenetically entirely unrelated to IpsD, as the former belongs to the PLP‐independent phosphatidylserine decarboxylase family that features a self‐cleavage mechanism to generate the prosthetic group necessary for Schiff base formation (Scheme S1).^[^ ^7^, ^10^ ^]^ The *ipsD* gene was heterologously expressed in *E. coli* to produce an N‐ and C‐biterminally tagged fusion protein, purified by metal affinity chromatography (Figure S1), and assayed for activity in vitro. Assuming decarboxylation represents the entry step for **1** biosynthesis in *I. corydalina* as well, we first tested IpsD with 1 mm **2** as substrate and analyzed for product formation by LC‐MS. Surprisingly, tryptamine (**6**) formation was not observed, and not even traces were detected (Figure 2a; Scheme 2), even after 16 h of incubation. This finding implies that IpsD does not accept **2** and, hence, cannot initiate the pathway.

In the previously elucidated **1** pathway, hydroxylation of the indole moiety *follows* decarboxylation. In contrast, mammalian serotonin formation involves ring hydroxylation *prior to* the decarboxylation step.^[^ ^31^, ^32^, ^33^ ^]^ We therefore tested whether **1** biosynthesis in *I. corydalina* follows the mammalian order of events and added 1 mm 4‐hydroxy‐l‐tryptophan (**7**) to the IpsD assay. The chromatographic analysis showed near‐quantitative turnover to **5** (*t*_R _= 2.1 min, *m*/*z* 177.1, [*M*+H]^+^ Figure 2a; Scheme 2), indicating that **1** biosynthesis may take a dissimilar course than in *Psilocybe* species (Scheme 1b). Still, decarboxylation must precede methylation in the cascade, as a primary amine is necessary for the decarboxylase catalytic cycle, during which an intramolecular hydrogen bond is formed between PLP and the substrate to establish a tautomeric system (Scheme S1).^[^ ^34^ ^]^

Kinetically, IpsD followed a typical Michaelis–Menten‐type response. We determined a *K*_M_ value of 66 µm and a *k*_cat_ of 0.44 s^−1^ which translates into a catalytic efficiency of *k*_cat_/*K*_M_ = 6.58 s^−1^ mm ^−1^ with **7** as substrate (Figure S2). This efficiency is more than two‐fold lower than that of *Psilocybe cubensis* PsiD (17.4 s^−1^ mm ^−1^)^[^ ^10^ ^]^ but comparable to that of CsTDC (*k*_cat_/*K*_M_ = 7.56 s^−1^ mm ^−1^).^[^ ^30^ ^]^

To date, only a low number of fungal tryptophan decarboxylases have been characterized for their substrate specificity. The PLP‐independent monomeric PsiD is unspecific and tolerates various ring substitutions.^[^ ^10^, ^19^, ^35^ ^]^ Likewise, the known PLP‐dependent aromatic amino acid decarboxylases CsTDC,^[^ ^30^ ^]^ and IasA*,^[^ ^36^ ^]^ are flexible and tolerate at least 5‐hydroxy‐l‐tryptophan as well. In contrast, a *Penicillium raistrickii* decarboxylase rejected 5‐hydroxy‐l‐tryptophan but curiously accepted phenylalanine and tyrosine.^[^ ^37^ ^]^

**Figure 3:**
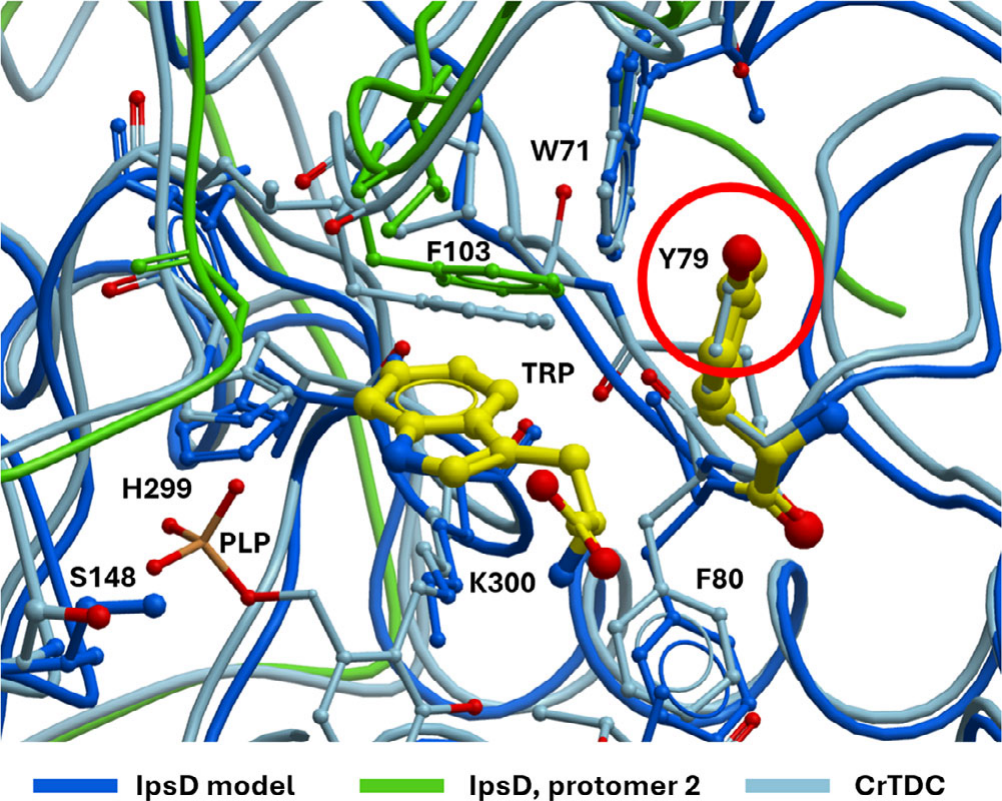
Structural superposition of the IpsD AlphaFold 3 model (dark blue) with CrTDC (PDB 6EEW, light blue). For the IpsD model, residues of the second protomer forming the binding site are displayed in green. The superposition is anchored at the *C*α of the PLP‐binding lysine residue K300 and shows additional key residues in the binding site (Figure S3). Significant is primarily the change in environment from the purely hydrophobic F100 around the substrate **2** in CrTDC to a polar component Y79 (red circle), which, given suitable conformational rearrangement, could stabilize the hydroxy group of **7**.

A decarboxylase that strictly rejects unsubstituted **2**, such as IpsD, is remarkable. To shed more light onto this phenomenon, we superimposed an IpsD model, created with AlphaFold 3 (Figure 3), on the crystal structure 6EEW of CrTDC, the l‐tryptophan decarboxylase of rose periwinkle (*Catharanthus roseus*).^[^ ^38^ ^]^ In contrast to monomeric PsiD,^[^ ^39^ ^]^ aromatic amino acid decarboxylases and the IpsD model form obligate dimers^[^ ^38^ ^]^ with buried interface surfaces of ∼6500 Å^2^.

The substrate binding pockets of CrTDC and IpsD are formed by residues from both protomers of the dimer (Figure S3). The PLP‐linked lysine residue in IpsD and the exactly superimposed CrTDC PLP‐lysine accurately anchor the superposition of the models and thus allow a plausible assessment of the binding sites given the bound **2** in the CrTDC structure (Figure 3). The substitution of CrTDC F100 with IpsD Y79 offers a plausible explanation as to why IpsD binds to 4‐hydroxylated but not to unsubstituted **2**: the additional hydroxy group generates a polar environment that allows, with structural relaxation, the formation of acceptor/donor interactions with the 4‐OH group, while at the same time disrupting the hydrophobic environment necessary to accommodate the aromatic indole group of the unsubstituted **2** (Figure 3). In summary, IpsD likely catalyzes the second biosynthetic step (Scheme 2) and contrasts the *Psilocybe* decarboxylase PsiD, which serves as a gatekeeping enzyme by catalyzing the initial step.

### Structural Model of the Monooxygenase IpsH

The second enzyme in the cascade familiar from *Psilocybe* species (Scheme 1b) is the cytochrome P_450_‐dependent monooxygenase PsiH that regioselectively introduces an oxygen atom to C‐4 of **6**. However, this verified monooxygenase^[^ ^7^ ^]^ and IpsH (Table S1), a putative cytochrome P_450_ enzyme as well, share only low sequence similarity (29.6% identical aa).

**Figure 4:**
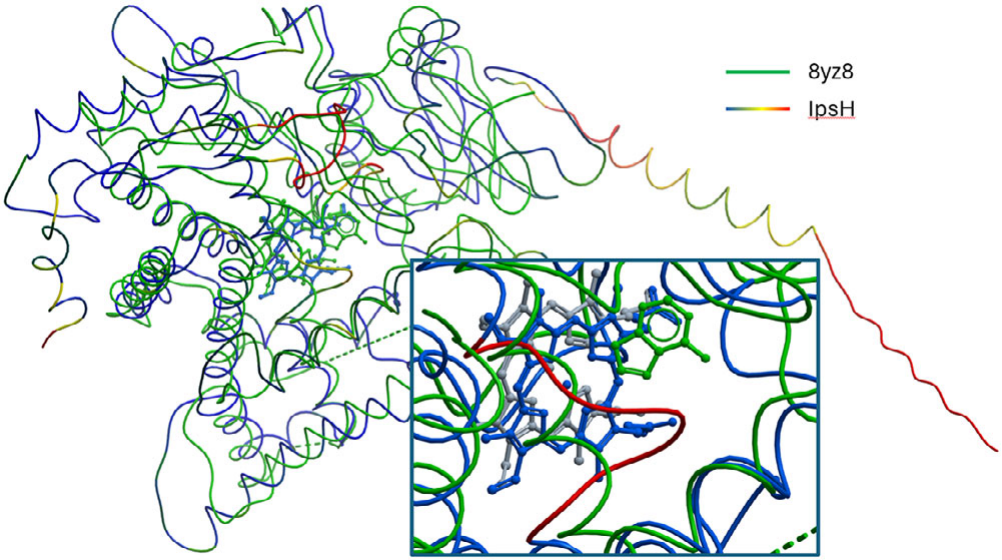
Structural superposition of the *I. corydalina* IpsH AlphaFold 3 model with 8YZ8. The IpsH backbone trace is colored by the B‐factor equivalent of the prediction reliability, from blue (high confidence) to red (low confidence). To the right, the extra N‐terminal extension of the IpsH membrane anchor is visible. The insert shows the bulge around G310 (red) that interrupts the continuous helix present in most cytochrome P_450_ monooxygenases and could be a distinct structural feature involved in IpsH substrate binding.

Like other P_450_ monooxygenases,^[^ ^40^ ^]^ IpsH possesses an N‐terminal helical membrane anchor which is clearly visible in an AlphaFold 3 in silico model (Figure 4). The prediction model of IpsH reveals that, in addition to the ∼30 residue long helical membrane anchor common to cytochrome P_450_ enzymes,^[^ ^40^ ^]^ an unstructured, approximately 13 residue long N‐terminal extension is present. Superposition with 8YZ8, a peroxygenase^[^ ^41^ ^]^ in complex with adenine as a placeholder for a potential **6** binding site, reveals an unusual insertion at G310 in the likely binding site that bulges out from a helix which is continuous in most P_450_ enzymes (Figure 4). This insertion covers the likely binding site and thus could play a role in **2** binding. Future crystal structures are required to confirm these predictions.

Most P_450_ monooxygenases are insoluble.^[^ ^40^ ^]^ Hence, IpsH was not available for in vitro assays, yet mechanistic reasons allow its placement as the gateway enzyme of the Ips pathway: Dissimilar to the *Psilocybe* pathway, the substrate specificity of IpsD precludes hydroxylation after the decarboxylation step. Furthermore, hydroxylation must precede phosphate ester formation, and decarboxylation cannot follow methylation, as the removal of the carboxy group requires a primary amine in the substrate (Schemes 2 and S1).

### Activity and Structural Model of the Kinase IpsK

The phosphoryloxy group of **1** is a very rare structural feature among natural products. In *P. cubensis*, the phosphate ester is introduced by the kinase PsiK that falls into the thioribokinase family.^[^ ^7^, ^11^ ^]^ IpsK (Table S1) shows a very low degree of sequence identity with PsiK of only 22.4% identical aa. A search in UniProt^[^ ^42^ ^]^ with IpsK as a query against characterized kinases did not return any further hits besides PsiK.

At this point, a first picture of the possible sequential enzymatic orders had emerged of how *I. corydalina* may assemble **1** (hydroxylation to **7** by IpsH, followed by IpsD‐catalyzed decarboxylation to **5** [Scheme 2]). The question remained how **1** formation is completed. To elucidate the late pathway steps, N‐terminally hexahistidine‐tagged IpsK (Figure S1) was assayed in vitro in TRIS‐HCl buffer, pH 7.5, with ATP (2 mm) as well as **5** (1 mm) as a phosphate acceptor substrate. In stark contrast to PsiK, LC‐MS analysis showed only minute amounts of **8** as an IpsK product (*t*_R _= 1.1 min, Figure 2b). To identify the authentic IpsK acceptor substrate after this surprising result, we next investigated other potential substrates. In separate assays, we investigated whether IpsK phosphorylates **7**, **10**, **4**, and **9** (Figure 2b).

Product formation was observed with all substrates except **7**. However, only **4** led to a quantitative turnover to **1** (*t*_R _= 3.2 min, *m*/*z* 285.1 [*M*+H]^+^ Figure 2b). This particular reaction is catalyzed by the kinase PsiK of the *P. cubensis* **1** pathway as well, yet does not fulfill a biosynthetic function there. Rather, it is considered a mechanism to protect the cells against the presence of free **4**, which may form oligomers that may unspecifically inactivate proteins.^[^ ^43^, ^44^ ^]^ With **7**, not even traces of a product were detected. Yet, the products **3** (*t*_R _= 1.7 min, *m*/*z* 271.1 [*M*+H]^+^) and aeruginascin (**11**, *t*_R _= 2.9 min, *m*/*z* 299.2 [*M*+H]^+^) resulted from **10** and **9** as respective substrates (Figure 2b; Scheme 2). Taking the pathway branch toward either **3** or **1** into account, the kinetic investigation of IpsK was carried out with either **4**, i.e., the dimethylated phosphate acceptor, or **10** as the monomethylated acceptor substrate. IpsK showed a clear preference for the dimethylated substrate **4**, evident by a *K*_M_ value of 20.5 µm for **4** versus 336.7 µm for **10**. The *k*_cat_ values were 0.69 s^−1^ for **4** and 0.37 s^−1^ for **10**, resulting in catalytic efficiencies of *k*_cat_/*K*_M_ = 33.51 and *k*_cat_/*K*_M_ = 1.09 s^−1^ mm ^−1^, respectively, for **4** and **10** (Figure S2).

**Figure 5:**
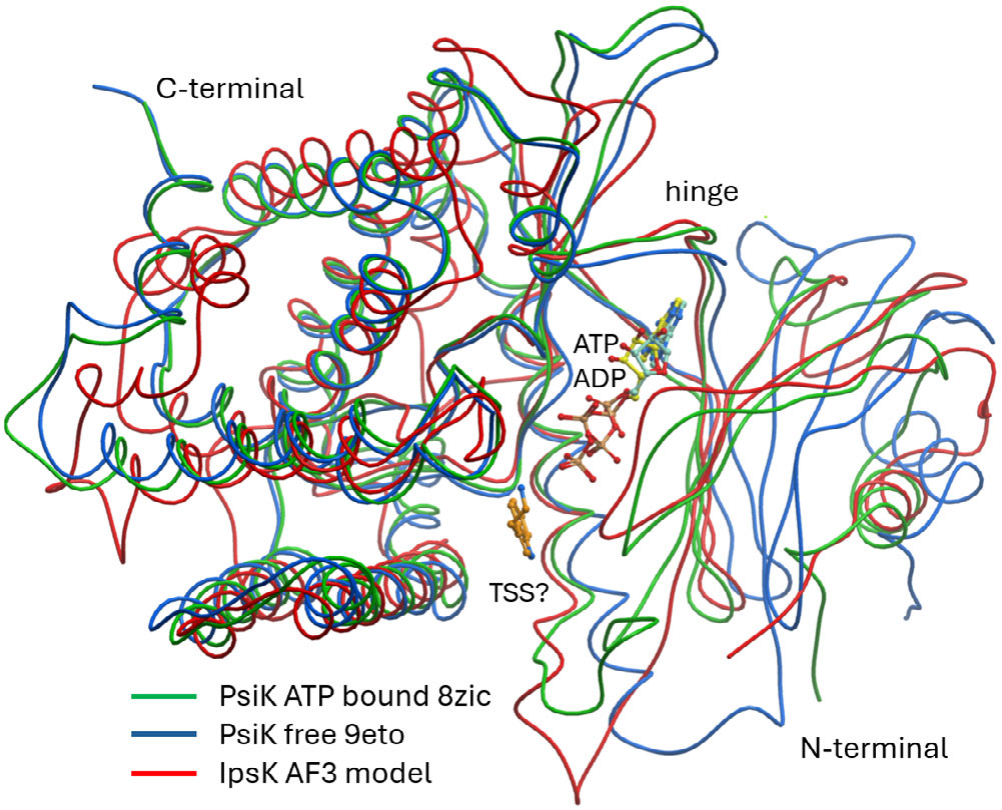
Structure comparison of PsiK versus IpsK. The C‐terminal domain (PsiK residues 122–362) aligns with 2.0 Å r.m.s.d. while the N‐terminal domains are in significantly different orientations in each of the three models. The unbound PsiK structure shows the largest opening of the binding site located between the N‐ and C‐terminal domains. The binding site with ADP/ATP is readily accessible in all models, and the ATP location in the prediction model agrees with the ADP‐bound X‐ray structure. The varying orientations of the respective N‐ and C‐terminal domains suggest that also in the case of IpsK, domain reorientation may play a crucial role during ATP/ADP loading/unloading and substrate processing. TSS stands for the PDB three‐letter code for tryptamine (**6**).

**Scheme 3:**
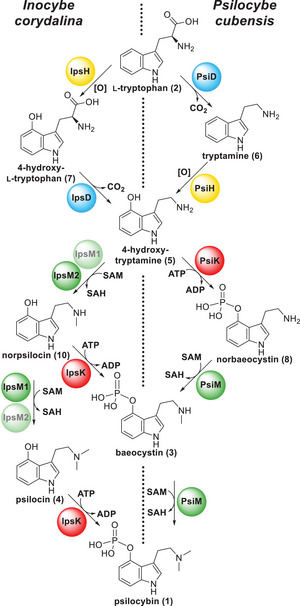
Biosynthetic pathways leading from **2** to **1** depicting the experimentally proven sequence and enzymes in *I. corydalina* (left) and *P. cubensis* (right). Note that **3** is not a substrate of any Ips enzyme. Hence, it is a final product in *I. corydalina*, whereas it represents a substrate for PsiM and, thus, the direct precursor to **1** in *P. cubensis*.

To explain the dissimilar substrate requirements of PsiK and IpsK, we modeled the IpsK structure in silico. Given that two X‐ray structures of PsiK are available, both were included in our comparison. The first PsiK structure that was reported (9ETO) is a polyethylene glycol (PEG)‐mediated dimer^[^ ^45^ ^]^ while a subsequently published model, 8ZIC contains ADP and **6** (TSS).^[^ ^39^ ^]^ The structure superposition of the two experimentally determined PsiK structures and the AlphaFold 3 model of IpsK is illustrated in Figure 5. Both IpsK and PsiK phosphorylate **4** to **1**, but IpsK exclusively requires methylated substrates. PsiK is indifferent to the methylation state, and its primary function in vivo is the phosphorylation of **5** to **8** (Scheme 3). Analysis of the binding pocket as to why IpsK hardly accepts the former with its primary amine side chain is difficult due to the ambiguous modelling of **6** in 8ZIC, which likely does not provide a good template for reliable modelling of the **4** substrate. The electron density for the **6**, modeled with partial occupancy in 8ZIC, is ambiguous. An alternative model of the ligand in 8ZIC with a PEG fragment placed into the same electron density (Figure S4) can be refined with higher real space correlation coefficients.^[^ ^46^ ^]^ PEG was present in high concentration in the crystallization cocktail^[^ ^39^ ^]^ and is then commonly observed in ligand binding sites.^[^ ^47^ ^]^

In summary, our biochemical results suggest IpsK as the final biosynthetic enzyme, which implies a reversed order of methylation and phosphotransfer compared to the situation in *Psilocybe*, where iterative methylation by a single enzyme, PsiM, completes the biosynthesis (Schemes 2 and 3).

### The Methyltransferase Pair IpsM1 and IpsM2

All known biosynthetic gene clusters in *Psilocybe* species code for only one methyltransferase, PsiM.^[^ ^22^, ^48^, ^49^ ^]^ In contrast, the published genomic sequence of *I. corydalina* encodes two near‐identical methyltransferases, IpsM1 and IpsM2 (93.1% identical aa, Table S1). These are members of the methyltransferase family 25 and are, hence, neither phylogenetically related to *Psilocybe* methyltransferases PsiM (for **1** biosynthesis) of family 10 nor to TrpM (*N*,*N*‐dimethyl‐l‐tryptophan synthase), which falls into family 33.^[^ ^50^ ^]^ AlphaFold 3 models of IpsM1 and IpsM2 show the typical Rossmann fold harboring the methyl source SAM. However, as a consequence of the phylogenetic distance, structural alignment with experimentally determined portions beyond the conserved SAM binding core is poor.

Neither the superposition with **8**‐bound PsiM (8PB4) nor a putative phosphoethanolamine *N*‐methyltransferase of *P. vivax* (4MWZ, unpublished) allowed meaningful predictions of substrate binding site details. Crystal structures of the enzyme complexes will be necessary to explain the differences between the methylation of the 4‐phosphorylated substrates **8** and **3** by PsiM versus the 4‐hydroxylated substrates **5** and **10** (IpsM2, IpsM1).

**Figure 6:**
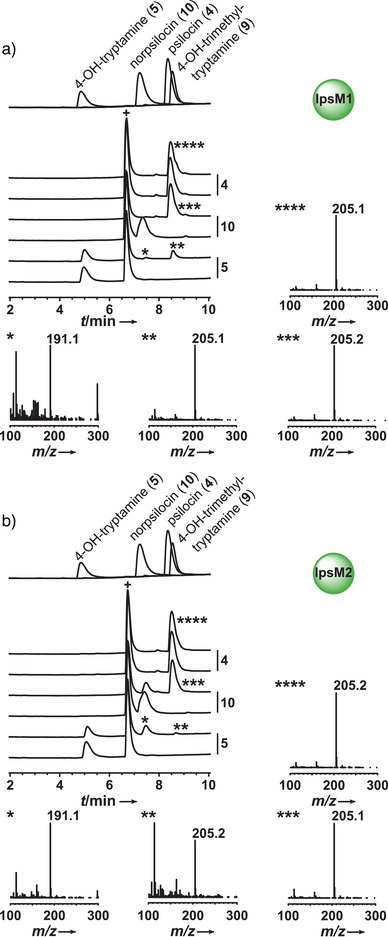
Chromatographic analysis with UV detection (λ = 280 nm) of in vitro product formation assays with a) methyltransferase IpsM1 and b) IpsM2 and the methyl donor SAM. Authentic standards are shown as overlaid individual chromatograms. The chromatogram pairs are designated with the respective substrates (**4**, **10**, and **5**). The bottom traces of each pair of chromatograms represent negative controls with heat‐treated enzymes; insets marked with asterisks depict mass spectra of the respective peak. Mass spectra were recorded in positive mode. The signal at *t*_R _= 6.7 min (marked with +) is the SAM degradation product 5′‐methylthioadenosine.^[^ ^51^, ^52^ ^]^ Reactions are shown in Scheme 2.

We addressed whether both enzymes are active and whether they catalyze a single or two consecutive methyl transfers. The heterologously produced and purified enzymes IpsM1 and IpsM2 (Figure S1) were assayed separately in vitro, again in TRIS‐HCl buffer, pH 7.5. The primary amine **5** was only poorly turned over by the kinase IpsK. Therefore, this compound appeared to be a more likely substrate for the two methyltransferases, which implies at least the first methyl transfer to precede the phosphotransfer. We offered **5**, but also **10**, as substrates (1 mm) in separate reactions. Both IpsM1 and IpsM2 were active, as shown by LC‐MS analyses (Figure 6), and the detected products pointed to similar catalytic activities. Both enzymes accepted **5** and catalyzed two consecutive methyl transfers, as evident by the products **4** and **10** (Scheme 2). However, we noticed quantitative differences of their ratio (Figure S5). While **4** was the major product of IpsM1 (*t*_R _= 8.6 min, *m*/*z* 205.1 [*M*+H]^+^), we detected mainly **10** in the IpsM2 assays (*t*_R _= 7.5 min, *m*/*z* 191.1 [*M*+H]^+^).

We compared the kinetics of these two methyltransferases, using both **5** and **10** as methyl acceptors. Even though the *K*_M_ values were comparable (101.0 µm for **5** and 138.2 µm for **10**), the *k*_cat_ values (0.08 s^−1^ for **5** and 0.19 s^−1^ for **10**) and the catalytic efficiencies (*k*_cat_/*K*_M_ = 0.83 s^−1^ mm ^−1^ for **5** and 1.40 s^−1^ mm ^−1^ for **10**) indicate a preference of IpsM1 for **10** (Figure S2). In contrast, the kinetic properties of IpsM2 showed an opposite preference: its *K*_M_ values were 64.5 µm for **5** but 433.2 µm for **10**. Likewise, we determined a *k*_cat_ = 0.11 s^−1^ for **5** and 0.08 s^−1^ for **10**, which leads to catalytic efficiencies of *k*_cat_/*K*_M_ = 1.72 s^−1^ mm ^−1^ for **5** and *k*_cat_/*K*_M_ = 0.18 s^−1^ mm ^−1^ for **10**.

Subsequently, we sought to confirm that IpsM1/IpsM2 reject phosphorylated substrates. We offered **8**, **3**, and **1**, i.e., the phosphorylated analogs of **5**, **10**, and **4**, in separate single‐enzyme assays as substrates. **1** was assayed for possible **11** formation (Scheme 2). None of these substrates led to product formation (Figure S6). This finding is congruent with the previous biochemical results on these two methyltransferases and provides additional evidence for the dissimilar sequential order of **1** assembly in *I. corydalina*, compared to the pathway order in *Psilocybe* species (Scheme 3).

### Multi‐Enzyme Activity Assays

**Figure 7:**
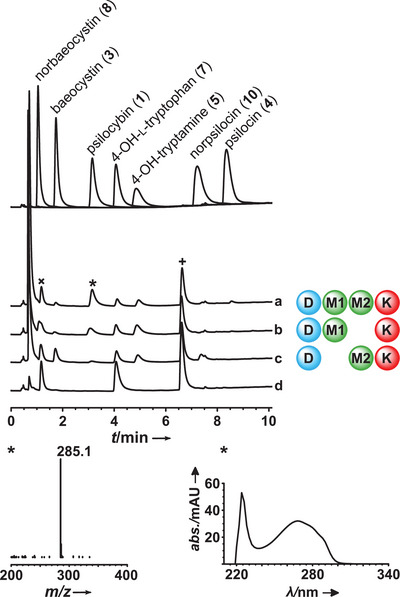
Chromatographic analysis with UV detection (λ = 280 nm) of multi‐enzyme in vitro product formation assays and **7** as substrate. Authentic standards are shown as overlaid individual chromatograms. Chromatogram a) reaction with IpsD, IpsM1, IpsM2, and IpsK; b) reaction with IpsD, IpsM1, and IpsK; c) reaction with IpsD, IpsM2, and IpsK; d) negative control with heat‐treated enzymes. The signal at *t*_R _= 6.7 min (+) is the SAM degradation product 5′‐methylthioadenosine.^[^ ^51^, ^52^ ^]^ The signal at *t*_R _= 1.2 min (×) was unconverted SAM and is not related to **8**, as evident by extracted ion chromatograms (Figure S7). Bottom left: mass spectrum (positive mode) of enzymatically synthesized **1**; bottom right: UV spectrum of **1**.

To gain a more profound understanding of if and how IpsM1 and IpsM2 cooperate, we ran multi‐enzyme assays, in all cases with **7** as substrate plus the required cofactors and co‐substrates PLP, ATP, SAM, and MgCl_2_. A one‐pot reaction containing the four enzymes IpsD, IpsK, IpsM1, and IpsM2 led to **1** formation (Figure 7), as expected from the outcome of the single‐enzyme assays.

Minor amounts of intermediates **5**, **4**, and **10**, along with the shunt product **3**, were also detected. Likewise, the triple‐enzyme combination IpsD, IpsK, and IpsM1 led to **1** formation as the main product as well, accompanied by **5**.

To some degree, the methyltransferases are redundant in that they turn over **5**, i.e., the chemically most unstable compound in the pathway.^[^ ^53^ ^]^ Still, following these in vitro results, the missing second methyltransferase (IpsM2) led to a quantitatively decreased and qualitatively shifted pathway product profile (Figure S8), pointing away from a simple scenario of two redundant methyltransferases fulfilling equal functions. This notion was supported by the opposite assay with IpsD and IpsK, but only IpsM2 for methyl transfer. In this combination, **3** became the major product, while **1** was only present in very modest titers (Figure 7). Intriguingly, **3** was found in equal or even higher titers than **1** in prior analytical works on the metabolite profile of *I. corydalina*.^[^ ^27^, ^54^, ^55^ ^]^ Of note, the pathway organization in *I. corydalina*, involving two semi‐redundant methyltransferases, makes both **3** and **1** end products of a branched biosynthesis (Scheme 2). Contrasting tertiary amine formation during **1** biosynthesis in *I. corydalina*, the two methyl transfer steps are catalyzed sequentially and iteratively by one single methyltransferase, PsiM, as the ultimate step in *P. cubensis*. Unlike in *I. corydalina*, **3** represents the direct precursor of **1** in *P. cubensis*, even though this second methyl transfer step is kinetically less favored than the first one.^[^ ^13^ ^]^

The term “convergent evolution” describes the independent appearance of similar morphological or physiological phenotypes in distantly or unrelated groups of organisms. For primary metabolism, a textbook example for this phenomenon is the assembly of l‐lysine in fungi along a pathway which is entirely unrelated to that in bacteria and plants.^[^ ^56^ ^]^ In the area of natural products of flowering plants, the pyrrolizidines^[^ ^57^ ^]^ or the benzoxazinoids^[^ ^58^ ^]^ are made along independently evolved pathways that rely, at least in part, on unrelated enzymes yet lead to the identical end product. Nitrogen‐containing betalains, or the pre‐anthraquinone pigments, emerged through parallel evolution in flowering plants or molds (ascomycetes) versus in mushrooms (basidiomycetes).^[^ ^59^, ^60^, ^61^ ^]^

However, convergently evolved pathways *within* the mushrooms—and even within the same mushroom order—have not been biochemically verified yet but surely raise the question of which particular environmental pressure made the mushrooms evolve a metabolic pathway to **1** and whether *I. corydalina* and *Psilocybe* species had been exposed to the same selective pressure. As a symbiont, the former follows a different lifestyle than *Psilocybe* species, which are saprotrophic wood‐ or dung‐inhabiting fungi that, consequently, inhabit different ecological niches. Previous research unambiguously established horizontal gene transfer as a major strategy to confer the capacity to produce **1** onto mushrooms.^[^ ^8^, ^21^, ^22^ ^]^ Other established mechanisms to evolve new metabolic capacities in fungi are vertical gene duplication^[^ ^62^ ^]^ and subsequent neofunctionalization, or de novo gene birth from previously non‐coding DNA.^[^ ^63^ ^]^ However, the evolutionary origin of the *ips* cluster remains unknown.

Building upon the hypothesis by Awan et al.,^[^ ^23^ ^]^ our results contribute biochemical evidence for a parallel **1**‐pathway evolution by recruiting and evolving a set of distantly or unrelated enzymes. Of note, outside the mushrooms, **1** was detected in cicada‐parasitizing evolutionary basal fungi (*Massospora* species).^[^ ^64^ ^]^ In these fungi unrelated to mushrooms, the biosynthesis genes have not been identified yet and may represent a third line of parallel evolution of **1** biosynthesis and the first instance outside the mushrooms.

Mutually non‐exclusive theories on the ecological function of **1** have been put forward and include modulation of the behavior of mycophagic predators, assuming neuroactive monomeric **4** is the ecologically relevant compound. Alternatively, oligo‐ and polymeric **4**, formed after mycelial damage, may represent an induced defense to deter predators due to protein‐inactivating effects.^[^ ^8^, ^44^, ^65^ ^]^ Intriguingly, the two **1** pathways found in mushrooms do not share any common reaction. While proceeding through **5** as the sole common intermediate, decarboxylation precedes regioselective cytochrome P_450_‐mediated introduction of an oxygen atom at *C*‐4 in *Psilocybe*, while these two early steps are reversed in *I. corydalina*. The two late steps follow an inverse sequence as well. Notably, the substrate spectrum of the kinase IpsK comprises **10**, making it less available for the second methyl transfer reaction (Schemes 2 and 3) but favoring accumulation of **3** in *I. corydalina* instead, as both methyltransferases reject phosphorylated compounds.

The enzyme triple of IpsK, IpsM1, and IpsM2 may hence constitute a finely balanced system that leads to **1** as long as both methyltransferases are active, yet allows the producer *I. corydalina* to shift the pathway toward **3** by downregulating (or otherwise attenuating) IpsM1. Our findings explain earlier reports that **3** was consistently and independently detected in *I. corydalina* fruiting bodies, besides **1**.^[^ ^27^, ^54^, ^55^ ^]^ Furthermore, the combined kinetic data support the concept of a kinetically regulated pathway branch: with the dimethylated substrate **4** to yield **1**, IpsK's catalytic efficiency is about 30 times higher than with monomethylated **10** which is converted to **3** (Scheme 3). IpsM1 is primarily catalyzing the second methyl transfer reaction and, thus, competes with IpsK for substrate **10**. The higher catalytic efficiency of IpsM1 with **10**, compared to IpsK, favors the branch toward **1**. In return, IpsK shows a higher catalytic efficiency with **10** than IpsM2. Consequently, in the absence of IpsM1, i.e., the second methyltransferase, the kinase IpsK may potentially outcompete IpsM2, and **3** would preferentially be formed. Therefore, the preference of IpsM1 for the monomethylated substrate **10** appears key to securing **1** production and is congruent with the findings with the coupled assays (Figures 7 and S8). Furthermore, the apparent role of IpsM2 is to supply **10** as a precursor.

The indolethylamine profile of *I. corydalina* has been exhaustively investigated.^[^ ^27^, ^54^, ^55^ ^]^ At the time of the first analyses,^[^ ^27^, ^54^ ^]^ **11** had not been described yet, whereas in the latest study, it was detected in traces.^[^ ^55^ ^]^ The substrate preferences of both methyltransferases and the IpsK kinase, experimentally determined in this work, are in part compatible with these prior analytical observations: IpsK phosphorylated **9** to **11**. However, based on our in vitro assays and using pure enzyme, this appears to be a non‐physiological reaction, as neither methyltransferase carries out a third methylation to establish a quaternary side chain ammonium necessary to provide IpsK with this particular substrate (Figures 7 and S6). Yet, **11** was detected in the eponymous sister species *I. aeruginascens* in higher quantities.^[^ ^66^ ^]^ Further research is warranted to address the biosynthetic enzymes, the number of methyltransferases, and their substrate specificities in the above‐mentioned species, for which genomic sequence data is not available yet, to elucidate the origin of this quaternary compound.

The indole alkaloid **1** is under consideration as a drug to treat therapy‐resistant depression.^[^ ^4^ ^]^ Advanced clinical trials and possibly approval and introduction into clinical use in various countries will entail an increasing demand. The discovery of the *psi* genes in *Psilocybe* species set the stage for heterologous production in surrogate hosts in vivo and in scalable cultures.^[^ ^15^, ^16^, ^17^, ^18^, ^20^ ^]^ In parallel, an in vitro procedure with immobilized enzymes constituting a reusable set of catalysts was recently devised.^[^ ^29^ ^]^ Regardless of the approach, the Ips enzymes, characterized in this work, contribute new enzymes to produce **1** biotechnologically and sustainably, which adds an applied component to our work.

## Conclusion

Our work contributes the biochemical foundation that **1** and **3** biosynthesis within the mushroom order Agaricales was selected twice independently, involving a set of enzymes with different substrate specificities, resulting in a different order of biosynthetic events. Probably the most intriguing question of natural product chemistry pertains to why natural products are made and what exact benefits they provide to the producers. As *Inocybe* and *Psilocybe* mushrooms follow different lifestyles, our work may help ecologists identify the selection pressure and true reason why one of the most iconic natural products emerged and why it emerged independently.

## Supporting Information

The authors have cited additional references within the Supporting Information.^[^ ^67^, ^68^, ^69^, ^70^, ^71^, ^72^, ^73^, ^74^, ^75^, ^76^, ^77^, ^78^, ^79^, ^80^, ^81^ ^]^

## Conflict of Interests

The authors declare no conflict of interest.

## Supporting information



Supporting Information

## Data Availability

The data that support the findings of this study are available in the Supporting Information of this article.
