# Supplementary material for: Dissimilar Reactions and Enzymes for Psilocybin Biosynthesis in Inocybe and Psilocybe Mushrooms
Source: Angew Chem Int Ed Engl. 2025 Sep 21;64(46):e202512017. doi: 10.1002/anie.202512017 (PMC12603985; doi:10.1002/anie.202512017)
Supplement: Supplementary file 1 — Supporting Information [file ANIE-64-e202512017-s001.pdf]

Dissimilar Reactions and Enzymes for Psilocybin Biosynthesis in *Inocybe* and *Psilocybe* Mushrooms

Tim Schäfer,[a,b] Fabian Haun,[a,b] Bernhard Rupp,[c,d] Dirk Hoffmeister*[a,b,e]

[a] T. Schäfer, F. Haun, Prof. Dr. D. Hoffmeister
Pharmaceutical Microbiology
Friedrich Schiller University
Winzerlaer Str. 2, 07745 Jena (Germany)
E-mail: dirk.hoffmeister@leibniz-hki.de

[b] T. Schäfer, F. Haun, Prof. Dr. D. Hoffmeister
Pharmaceutical Microbiology
Leibniz Institute for Natural Product Research and Infection Biology - Hans-Knöll-Institute
Beutenbergstrasse 11a
07745 Jena (Germany)

[c] Univ.-Doz. Mag. Dr. B. Rupp,
Department of General, Inorganic and Theoretical Chemistry
University of Innsbruck
Innrain 82
6020 Innsbruck (Austria)

[d] Univ.-Doz. Mag. Dr. B. Rupp,
k.k. Hofkristallamt
San Diego, CA (USA)

[e] Prof. Dr. D. Hoffmeister
Cluster of Excellence Balance of the Microverse
Friedrich Schiller University
Neugasse 23, 07743 Jena (Germany)

Table of Contents

Figure S1. SDS polyacrylamide gel electrophoresis of purified His_6-tagged enzymes 2
Figure S2. Kinetic characterization of Ips enzymes. 3
Figure S3. Comparison of key residues in the binding pocket of decarboxylases. 4
Figure S4. Binding pocket of the kinase PsiK, used for IpsK analysis. 5
Figure S5. Relative quantification of substrate and products in assays with IpsM1 and IpsM2. 6
Figure S6. *In vitro* assays with methyltransferases IpsM1 and IpsM2 and phosphorylated substrates. . 7
Figure S7. Extracted ion chromatograms of multi-enzyme assays. 8
Figure S8. Relative quantification of products of the multi-enzyme assays.. 9

Scheme S1. PLP-dependent and PLP-independent decarboxylation 10

Table S1. Basic characteristics of psilocybin biosynthetic genes and enzymes in *Inocybe corydalina*. 11
Table S2. Comparison of psilocybin biosynthetic enzymes. 12
Table S3. Characteristics of psilocybin biosynthetic genes and enzymes in *Inocybe corydalina*. 12

Experimental Section 13

References 15

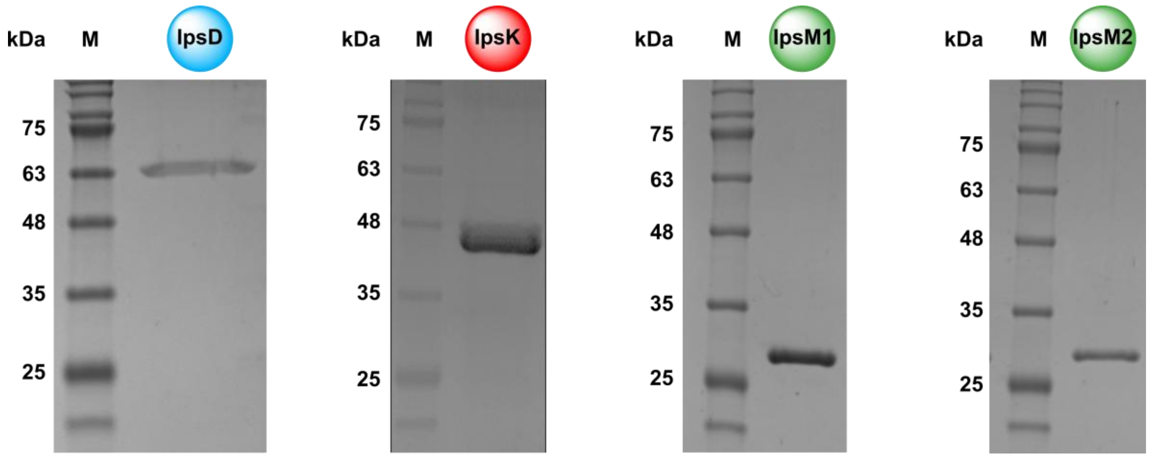

Figure S1. SDS polyacrylamide gel electrophoresis of purified His$_6$-tagged *Inocybe corydalina* enzymes. The calculated masses of hexahistidin-tagged IpsD, IpsK, IpsM1, and IpsM2 are 61.6, 45.4, 30.4, and 30.2 kDa, respectively. M: Protein standard (BlueEye pre-stained marker, Jena Bioscience).

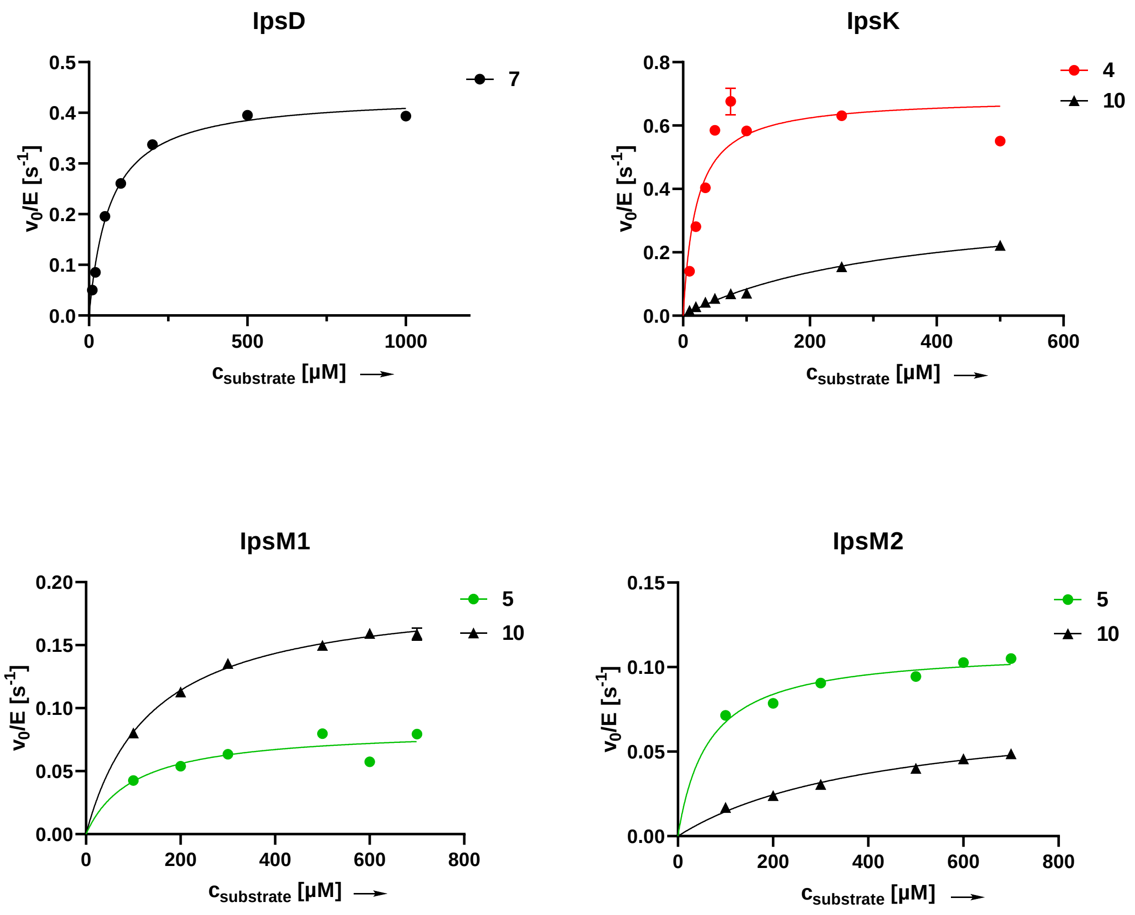

Figure S2. Kinetic characterization of Ips enzymes. Shown are plots for initial velocities for

- 4-hydroxy-L-tryptophan (**7**) with IpsD (top left),
- psilocin (**4**) and norpsilocin (**10**) with IpsK (top right),
- 4-hydroxytryptamine (**5**) and **10** with IpsM1, and
- and **5** and **10** with IpsM2.

Error bars smaller than the data point are not shown.

IpsD #	71A	79A	80A	81A	82A	101B	103B	148B	239A	299A	300A	306A	350B	351B
IpsD AA	W	Y	F	P	A	P	F	S	T	H	K	F	L	G
CrTDC AA	W	F	F	P	A	V	F	S	T	H	K	L	T	G
CrTDC #	92A	100A	101A	102A	103A	122B	124B	168B	262A	318A	319A	325A	369B	370B

Figure S3. Comparison of key residues in the binding pocket of decarboxylases. Aromatic amino acid decarboxylases CrTDC and IpsD were aligned, based on the superposition of the AlphaFold 3 model of IpsD with the experimentally determined structure of CrTDC (PDB 6EEW). Blue-shaded residues belong to the chain A of the dimers, green-shaded residues belong to chain B. The pyridoxal-5'-phosphate (PLP)-binding lysine residue is shown in red in a yellow background. The single relevant difference in the tryptophan (**2**) binding region is the exchange of phenylalanine 100 in CrTDC versus tyrosine 79 in IpsD.

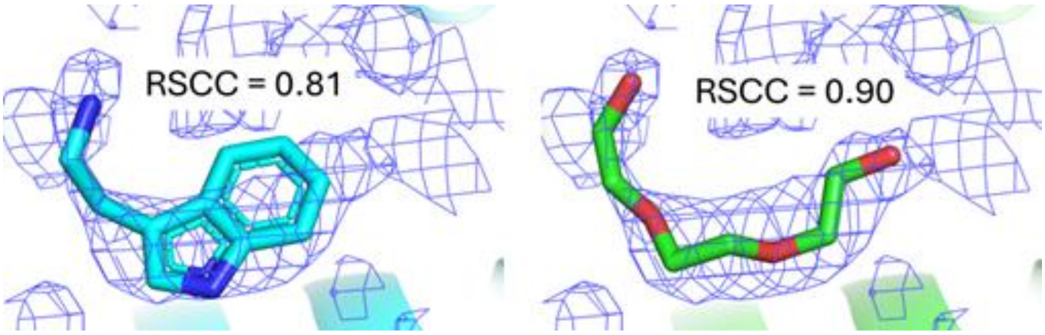

Figure S4. Binding pocket of the kinase PsiK, used for IpsK analysis. The comparison of the electron density map (blue grid) and the real space correlation coefficient (RSCC) of tryptamine (**6**) and a polyethylene glycol fragment (ball and stick model). The 2mFo-DFc map calculated from the deposited data of entry 8ZIC is contoured at a 0.8 σ.

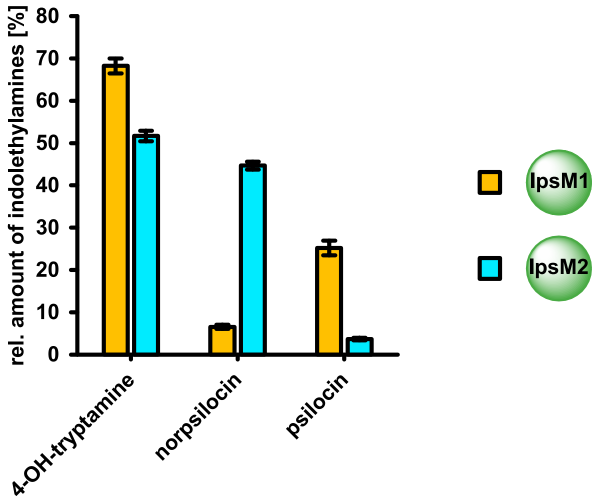

Figure S5: Relative quantification of substrate and products in single-enzyme assays with IpsM1 and IpsM2. *In vitro* product formation with 4-hydroxytryptamine (**5**) as substrate and IpsM1 (yellow bars) or IpsM2 (cyan bars) is shown. Error bars represent the standard deviation. Sample size n = 3.

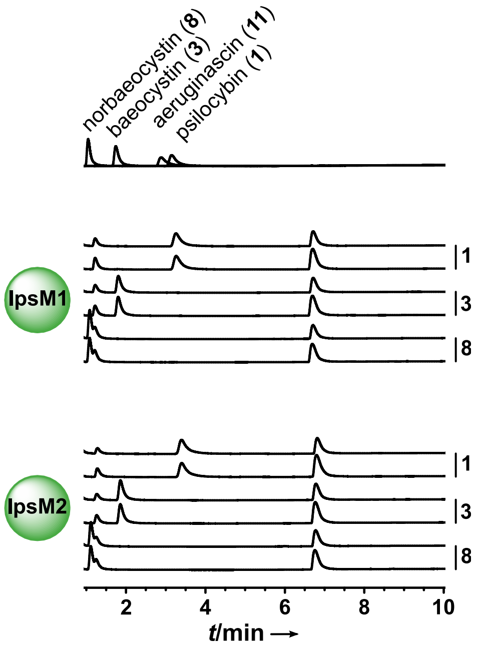

Figure S6. ***In vitro*** **product formation assays with methyltransferases IpsM1 and IpsM2.** The methyl donor substrate was *S*-adenosyl-L-methionine (SAM). Shown are chromatograms recorded by UV detection (λ = 280 nm). Authentic standards are shown as overlaid individual chromatograms (top). Pairs of chromatograms are designated by the respective substrates: psilocybin (**1**); baeocystin (**3**); norbaeocystin (**8**). None of these substrates led to detectable product formation. Bottom traces of each pair of chromatograms represent negative controls with heat-treated enzymes. The signal at t_R=6.7 min is the SAM degradation product 5′-methylthioadenosine.[51,52]

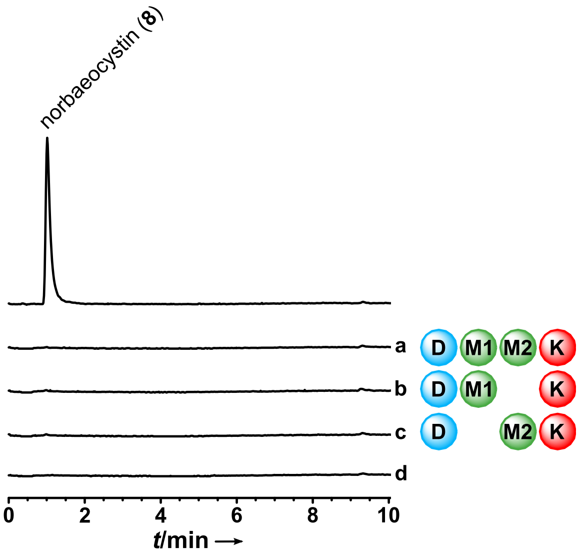

Figure S7. Extracted ion chromatograms of multi-enzyme assays. *In vitro* product formation reactions with 4-hydroxy-L-tryptophan (**7**) as substrate were analyzed by UHPLC-MS, and EICs were extracted to detect norbaeocystin (**8**, *m*/*z* 257 [*M* + H]$^+$),

Top chromatogram: Extracted ion chromatogram of **8**. Chromatogram a) reaction with IpsD, IpsM1, IpsM2, and IpsK; b) reaction with IpsD, IpsM1, and IpsK; c) reaction with IpsD, IpsM2, and IpsK; d) negative control with heat-inactivated enzymes IpsD, IpsM1, IpsM2, and IpsK.

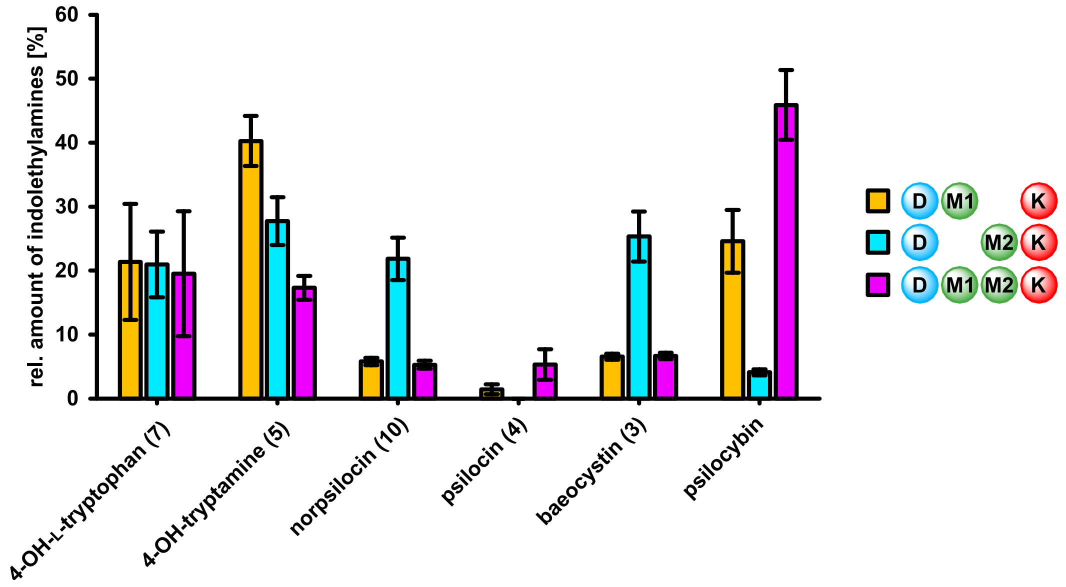

Figure S8. Relative quantification of products of the multi-enzyme assays. *In vitro* product formation reactions with 4-hydroxy-L-tryptophan (**7**) and enzyme combinations IpsD, IpsM1, and IpsK (orange bars), IpsD, IpsM2, and IpsK (cyan bars), and IpsD, IpsM1, IpsM2, and IpsK (magenta bars). Error bars represent the standard deviation. Sample size n = 3.

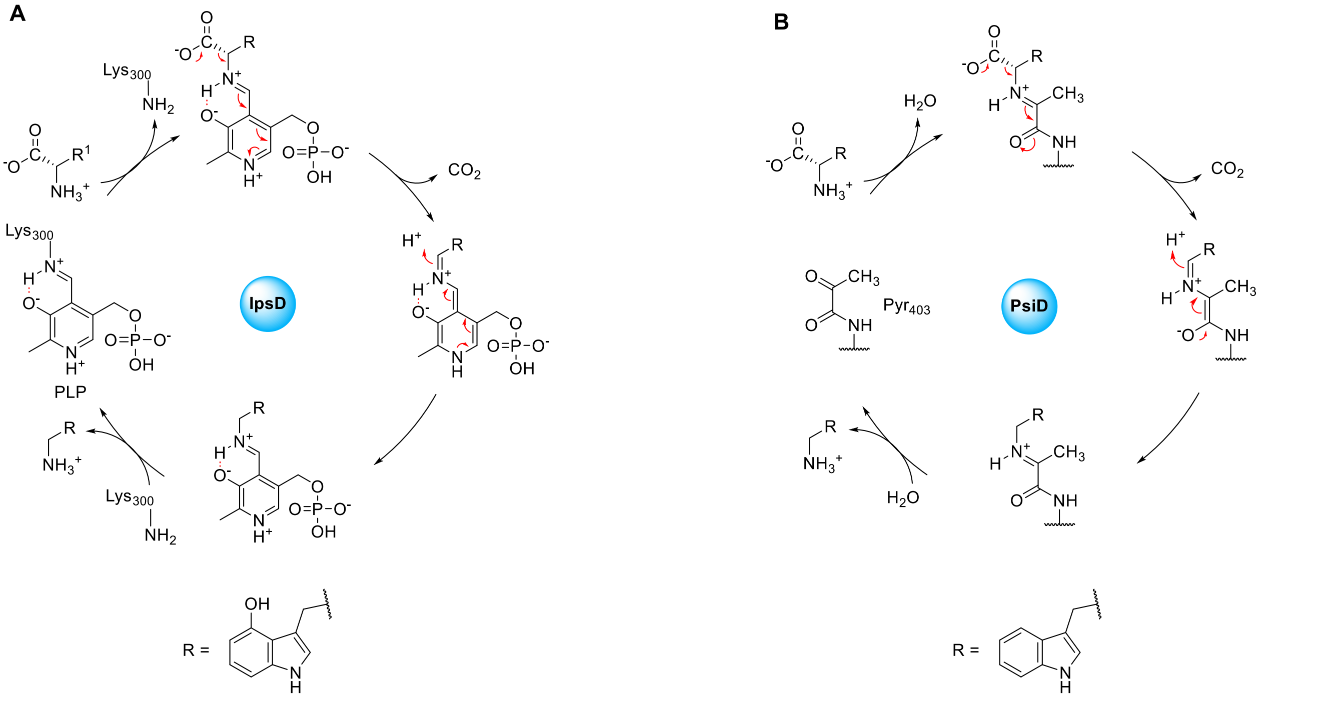

Scheme S1. PLP-dependent and PLP-independent decarboxylation. Comparison of the decarboxylation mechanisms by A) *I. corydalina* IpsD and B) *P. cubensis* PsiD during psilocybin (**1**) biosynthesis. Note the shared principle of delocalized electrons at the *C*α of the substrate amino acid in PLP-dependent (IpsD) and pyruvoyl-dependent (PsiD) decarboxylases. The proton at the amino group plays a crucial role in both catalytic mechanisms, which explains why only α-amino acids with primary amines are amenable to catalytic decarboxylation by PLP- and pyruvoyl-dependent decarboxylases.

Table S1. Basic characteristics of psilocybin biosynthetic genes and enzymes in *Inocybe corydalina*. The function of the *ipsH* gene product is predicted, all other functions have been verified experimentally. Molecular masses of enzymes are calculated.

Gene	Length [bp]	Number of introns	cDNA length [bp]	Function of encoded enzyme	Genbank accession #
ipsH	1986	8	1503	monooxygenase	PV569110
ipsD	2033	10	1506	4-OH-L-Trp decarboxylase	PV569111
ipsK	1203	1	1146	kinase	PV569112
ipsM1	738	0	735	*N*-methyltransferase	PV591345
ipsM2	738	0	735	*N*-methyltransferase	PV591346

Enzyme	Length [aa]	Molecular mass (native) [Da]	pI (calc.)	Molecular mass (including tag) [Da]	Position of hexa-histidine tag
IpsH	501	56,140	8.65	-	-
IpsD	502	56,503	5.72	61,567	N- and C-terminal
IpsK	382	41,929	6.52	45,472	N-terminal
IpsM1	245	26,819	4.56	30,363	N-terminal
IpsM2	245	26,647	4.56	30,191	N-terminal

Table S2. Comparison of psilocybin biosynthetic enzymes. Shown are *Inocybe corydalina* enzymes (this work) and their functional equivalents of *Psilocybe cubensis*.[7] The function of all enzymes has been verified experimentally, except IpsH, whose function has been deduced from sequence comparisons and by structural modeling.

Inocybe corydalina		***Psilocybe cubensis***	
**Enzyme**	**Enzyme family**	**Enzyme**	**Enzyme family**
IpsH	Cytochrome P_{450} family 64	PsiH	Cytochrome P_{450} family 64
IpsD	DOPA decarboxylase	PsiD	Phosphatidylserine decarboxylase
IpsM1	Methyltransferase family 25	PsiM	Methyltransferase family 10
IpsM2	Methyltransferase family 25		
IpsK	YcbJ superfamily	PsiK	YcbJ superfamily

Table S3. Sequence similarities of *Inocybe corydalina* psilocybin biosynthetic enzymes. Using the Ips enzymes as query, a blastP[67,68] search was conducted in non-redundant sequence database mode to identify the best hit (lowest e-value). Furthermore, a UniProt[42] search was carried out to find the closest experimentally verified enzyme. Except *Psilocybe cyanescens*, none of the organisms listed in this table is known to produce psilocybin (**1**). The dissimilarity between the *I. corydalina* and *P. cubensis* enzymes is shown by a pair-wise sequence alignment of corresponding biosynthetic enzymes (Table S2), using ClustalW[69] (BLOSUM matrix) implemented in Geneious software.

**Enzyme**	**BlastP results (identical/similar aa in %)**	**UniProt hit (identical/similar aa in %)**	***P. cubensis* enzyme (id./similar aa in %)**
IpsH	RDB27330.1 *Hypsizygus marmoreus* (62/77)	F1SY66.1 *Postia placenta* (48/65)	PsiH 26.5/44.4
IpsD	KAF8070404.1 *Lyophyllum atratum* (72/85)	Q7XHL3.1 *Oryza sativa* (42/61)	PsiD 9.0/24.4
IpsM1	HEY7590680.1 c*and. Limnocylindrales bacterium* (43/56)	A3QEP9.1 *Shewanella loihica* (33/51)	PsiM 14.7/26.7
IpsM2	MCJ1354669.1 *Icmadophila ericetorum* (39/53)	A3QEP9.1 *Shewanella loihica* (34/52)	PsiM 13.5/31.2
IpsK	KIM77107.1 *Piloderma croceum* (40/57)	A0A286LEZ6.1 *Psilocybe cyanescens* (24/39)	PsiK 19.7/33.7

Experimental Section

Materials and general procedures. Chemicals, solvents, and media ingredients were purchased from AstaTech, Roth, Sigma-Aldrich, and VWR. Synthesized 4-hydroxytryptamine (**5**), norbaeocystin (**8**) and baeocystin (**3**) were provided by Dr. Claudius Lenz (Friedrich Schiller University Jena) and Alexander Sherwood, Ph.D. (Usona Institute, Madison, WI), norpsilocin (**10**) was provided by Markus Winkler and Professor Dr. Peter Spiteller (University of Bremen), 4-hydroxy-*N*,*N*,*N*-trimethyltryptamine (**9**) was provided by Professor David Manke, Ph.D. (University of Massachusetts, Dartmouth, MA). Plasmid isolation, DNA restriction, and ligation followed the instructions of the manufacturers of kits and enzymes (Macherey-Nagel, NEB, Promega). For heterologous production of biosynthetic enzymes, *Escherichia* (*E.*) *coli* KRX (Promega) was used.

Bioinformatic methods. The published genomic sequence data[23] was used to create a local blast[67,68] database using Geneious (7.1.9) software. The biosynthetic gene cluster postulated before was located using the described amino acid sequences as query for a tblastn search. Introns in the biosynthetic genes were predicted using the Augustus[70,71] algorithm and manually edited in the case of *ipsH*. Sequence similarities were searched using blastP[67,68] and UniProt.[42] The coding sequences of the Ips enzymes are deposited under GenBank accession numbers PV569110 (*ipsH*), PV569111 (*ipsD*), PV569112 (*ipsK*), PV591345 (*ipsM1*), and PV591346 (*ipsM2*).

Construction of expression plasmids. The synthetic genes *ipsD, ipsM1, ipsM2 and ipsK* were individually ligated to the *Bam*HI/*Hin*dIII co-restricted expression vector pET28a, using the Gibson method[72] and the NEBuilder Hifi DNA Assembly Kit (NEB). *E. coli* KRX was transformed with the resulting constructs pTS127 (*ipsD*), pTS128 (*ipsM1*), pTS129 (*ipsM2*), pTS130 (*ipsK)*, and pFB15[50,73] (encoding *S*-Adenosyl-L-homocysteine nucleosidase MtnN), respectively.

Heterologous production of IpsD, IpsM1, IpsM2, IpsK, and MtnN. The transformed *E. coli* strains production cultures (2 × YT liquid medium plus 50 µg mL^{-1} kanamycin, 500 mL dispensed in 2 L Erlenmeyer flasks) were inoculated with a 5 mL overnight LB starter culture and incubated at 180 rpm and 37 °C until it reached an optical density of OD_{600} = 0.6. Cells were induced with 0.1% (m/v) L-rhamnose and 1 mM IPTG, shifted to 16 °C and 180 rpm, and incubated for another 20 h before being harvested. Cultures were centrifuged (3,220 × g, 4 °C, 30 min), the supernatant was discarded and the pellet was resuspended in 7 mL lysis buffer (10 mM imidazole in 50 mM sodium phosphate buffer, pH 8, also containing 300 mM NaCl) per liter culture. Hexahistidine-tagged enzymes were purified by immobilized metal affinity chromatography as described.[10] The enzymes were rebuffered into TRIS-HCl buffer (pH 7.5, 50 mM), using Cytiva PD-10 columns. The protein concentrations were determined by Bradford's assay.[74]

Single-enzyme *in vitro* product formation assays*.* The reactions were set up in reaction buffer (TRIS-HCl, pH 7.5, 50 mM, amended with 3 mM sodium thiosulfate) in a total volume of 50 µL, 0.5 µM enzyme, and were incubated overnight (16 h) at room temperature. Decarboxylase assays contained 0.1 mM pyridoxal 5'-phosphate (PLP), and 1 mM of substrate (**7** or **2**). The methyltransferase assays contained 4 mM *S*-adenosyl-L-methionine (SAM) and 1 mM substrate (**5**, **10**, **4**, **8**, **3**, and **1**), and 2 µM *S*-adenosyl-L-homocysteine (SAH) nucleosidase (MtnN). Kinase assays contained 2 mM ATP sodium salt, 1 mM $MgCl_2$ and 1 mM substrate (**7**, **5**, **10**, **4**, or **9**). Heat-inactivated enzyme served as negative control and the reactions were run in triplicate. After the incubation, reactions were frozen lyophilized, resuspended in 100 µL methanol, centrifuged, and 1 µL of the supernatant was subjected to liquid chromatography.

Enzyme kinetics were recorded at room temperature, all assays were run in triplicate. IpsD assays contained 200 nM enzyme, 0.1 mM PLP and 10 to 1000 µM **7** in 50 mM TRIS-HCl buffer (pH 7.5). IpsM1 and M2 assays contained 250 nM enzyme, 1 mM SAM, 2 µM MtnN and 100 to 700 µM **5** or **10** in 50 mM TRIS-HCl buffer (pH 7.5). IpsK kinetic assays contained 100 nM IpsK when substrate **4** was present, or 500 nM IpsK for substrate **10**, furthermore 1 mM ATP, 1 mM $MgCl_2$ and 10 to 500 µM **4** or **10** in 50 mM TRIS-HCl buffer (pH 7). The products were quantified using a calibration line of synthetic standards as reference.

Multi-enzyme *in vitro* product formation assay*.* The assay combining IpsD, IpsM1 and/or IpsM2, as well as IpsK was set up with 0.5 µM of each enzyme, 1 mM of **7**, 0.1 mM PLP, 4 mM SAM, 2 mM ATP, 1 mM $MgCl_2$ and 2 µM SAH nucleosidase MtnN. All other conditions were taken over from the single-enzyme assays.

Analytical liquid chromatography and mass spectrometry. To analyze *in vitro* assays, an Agilent 1290 Infinity II UHPLC-MS instrument was used which was fitted with a diode array detector and interfaced to a 6130 quadrupole mass detector, operated in positive mode and using electrospray ionization. The chromatograph was equipped with a Supelco Ascentis Express F5 column (100 × 2.1 mm, 2.7 µm particle size) and a guard column. Solvent A was 0.1% aqueous formic acid (FA), solvent B was methanol. For IpsD assays and kinetics, the column was kept at 50 °C. The solvent flow was 0.5 mL min^{-1}. A linear gradient of initially 10% B and within 8 min to 100% was applied. To analyze IpsK, IpsM1, IpsM2, and multi-enzyme assays, the column was maintained at 35 °C. A linear gradient was applied (% B): 0–4 min 2% at a flow of 0.6 mL min^{-1}. Within further 10 min, B was increased to 100% with a linear decrease of the flow to 0.4 mL min^{-1}.

Chromatographic analysis of IpsK, IpsM1, and IpsM2 kinetic assays and corresponding calibrations, were carried out on the chromatograph described above but a Phenomenex Luna Omega Polar C18 column (50 × 2.1 mm, 1.6 µm particle size) and an appropriate guard column. Separations were performed at 25°C and a flow of 0.5 mL min^{-1}. Solvent A was 0.1% aqueous FA, solvent B was acetonitrile. A linear gradient was applied (% B): initially 1%, within 3 min to 5%, then within 1 min to 100%.

***In silico* modelling and structural analysis of Ips enzymes.** Models of the *I. corydalina* Ips enzymes were obtained submitting their translated coding sequences[23] to the AlphaFold 3 (AF3) server,[75] using default settings. The respective ligands or cofactors were co-modelled (IpsH, heme; IpsK, ATP) or their location determined by SSM (Secondary Structure Matching) superposition[76] of the enzymes with available X-ray structures (IpsD, PLP, 6EEW). For comparison with experimental X-ray

structures, the Local Distance Difference Test (LDDT) quality measures of the models were converted to B-factor equivalents.[77] The respective coordinate sets of five models were identical within few tenths of an Å (IpsD, 0.21(04); IpsH, 0.68(13); IpsK, 0.42(08). For analysis and comparative illustrations, the highest scoring model was used. SSM coordinate r.m.s.d.s in Å between the respective models and experimental structure models were IpsD-6EEW_A: 1.8; IpsH-8YZ8_A: 2.5; IpsK-8ZIC: 2.1. The alternative 8ZIC model with a PEG fragment was built in *COOT*[78] and refined with *REFMAC5.*[79] Real space map correlation coefficients were calculated via default Polder maps in *Phenix*.[80] Dimer interfaces (IpsD, 6EEW) were analysed using PDBePisa.[81]

References

[7] J. Fricke, F. Blei, D. Hoffmeister, *Angew. Chem. Int. Ed*. **2017**, *56*, 12352–12355, *Angew. Chem.* **2017**, *129*, 12524–12527.

[10] T. Schäfer, K. Kramer, S. Werten, B. Rupp, D. Hoffmeister, *ChemBioChem* **2022**, *23*:e202200551.

[23] A. R. Awan, J. M. Winter, D. Turner, W. M. Shaw, L. M. Suz, A. J. Bradshaw, T. Ellis, B. T. M. Dentinger, *BioRxiv*, **2018**, doi: https://doi.org/10.1101/374199.

[42] The UniProt Consortium, *Nucleic Acids Res*. **2025**, *53*, D609–D617.

[50] F. Blei, J. Fricke, J. Wick, J. C. Slot, D. Hoffmeister, *ChemBioChem* **2018**, *19*, 2160–2166.

[51] D. F. Iwig, S. J. Booker, *Biochemistry* **2004**, *43*, 13496–13509.

[52] T. D. Huber, F. Wang, S. Singh, B. R. Johnson, J. Zhang, M. Sunkara, S. G. Van Lanen, A. J. Morris, G. N. Phillips, J. S. Thorson, *ACS Chem. Biol*. **2016**, *11*, 2484–2491.

[67] S. F. Altschul, W. Gish, W. Miller, E. W. Myers, D.J. Lipman, *J. Mol. Biol*. **1990**, *215*, 403–410.

[68] S. F. Altschul, T. L. Madden, A. A. Schaffer, J. Zhang, Z. Zhang, W. Miller, D. J. Lipman, *Nucleic Acids Res*., **1997**, *25*, 3389–3402.

[69] J. D. Thompson, D. G. Higgins, T. J. Gibson, *Nucleic Acids Res*., **1994**, *22*, 4673–4680.

[70] M. Stanke, O. Schöffmann, B. Morgenstern, S. Waack, BMC *Bioinformatics*, **2006**, *7*: 62.

[71] M. Stanke, B. Morgenstern, *Nucleic Acids Res*. **2005**, *33*, W465–W467.

[72] D. G. Gibson, L. Young, R.-Y. Chuang, J. C. Venter, C. A Hutchison III, H. O. Smith, *Nat. Methods*, **2009**, *6*, 343–345.

[73] A. Vit, L. Misson, W. Blankenfeldt, F. P. Seebeck, *ChemBioChem* **2015**, *16*, 119–125.

[74] M. M. Bradford, *Anal. Biochem*. **1976**, *72*, 248–254.

[75] J. Abramson, J. Adler, J. Dunger, R. Evans, T. Green, A. Pritzel, O. Ronneberger, L. Willmore, A. J. Ballard, J. Bambrick, S. W. Bodenstein, D. A. Evans, C. C. Hung, M. O'Neill, D. Reiman, K. Tunyasuvunakool, Z. Wu, A. Zemgulyte, E. Arvaniti, C. Beattie, O. Bertolli, A. Bridgland, A. Cherepanov, M. Congreve, A. I. Cowen-Rivers, A. Cowie, M. Figurnov, F. B. Fuchs, H. Gladman, R. Jain, Y. A. Khan, C. M. R. Low, K. Perlin, A. Potapenko, P. Savy, S. Singh, A. Stecula, A. Thillaisundaram, C. Tong, S. Yakneen, E. D. Zhong, M. Zielinski, A. Zidek, V. Bapst, P. Kohli, M. Jaderberg, D. Hassabis, J. M. Jumper, *Nature*, **2024**, *630*, 493–500.

[76] E. Krissinel, K. Henrick, *Acta Crystallogr*. **2004**, *D60*, 2256–2268.

[77] R. D. Oeffner, T. I. Croll, C. Millan, B. K. Poon, C. J. Schlicksup, R. J. Read, T. C. Terwilliger, *Acta Crystallogr D*, **2022**, *D78*, 1303–1314.

[78] J. E. Debreczeni, P. Emsley, *Acta Crystallogr.* **2012**, *D68*, 425–430.

[79] G. N. Murshudov, P. Skubak, A. A. Lebedev, N. S. Pannu, R. A. Steiner, R. A. Nicholls, M. D. Winn, F. Long, A. A. Vagin, *Acta Crystallogr.* **2011**, *D67*, 355–367.

[80] D. Liebschner, P. V. Afonine, M. L. Baker, G. Bunkoczi, V. B. Chen, T. I. Croll, B. Hintze, L.-W. Hung, S. Jain, A. J. McCoy, N. W. Moriarty, R. D. Oeffner, B. K. Poon, M. G. Prisant, R. J. Read, J. S. Richardson, D. C. Richardson, M. D. Sammito, O. V. Sobolev, D. H. Stockwell, T. C. Terwilliger, A. G. Urzhumtsev, L. L. Videau, C. J. Williams, P. D. Adams, *Acta Crystallogr.* **2019**, *D75*, 861–877.

[81] E. Krissinel, K. Henrick, *J. Mol. Biol*. **2007**, *372*, 774–797.
